# Research progress of *Gastrodia elata* Blume polysaccharides: a review of chemical structures and biological activities

**DOI:** 10.3389/fchem.2024.1395222

**Published:** 2024-07-02

**Authors:** Liu Yang, Shi-Hui Qin, Cheng-Ting Zi

**Affiliations:** ^1^ State Key Laboratory of Quality Research in Chinese Medicine, Macau Institute for Applied Research in Medicine and Health, Macau University of Science and Technology, Taipa, Macao SAR, China; ^2^ State Key Laboratory of Phytochemistry and Plant Resources in West China, Kunming Institute of Botany, Chinese Academy of Sciences, Kunming, China; ^3^ Research Center for Agricultural Chemistry, College of Science, Yunnan Agricultural University, Kunming, China; ^4^ Key Laboratory of Pu-erh Tea Science, Ministry of Education, College of Food Science and Technology, Yunnan Agricultural University, Kunming, China

**Keywords:** *Gastrodia elata*, polysaccharides, chemical structure, pharmacological activity, mechanism

## Abstract

*Gastrodia elata* Blume (*G. elata*), listed as one of the 34 precious Chinese medicines, servers a dual purpose as both a medicinal herb and a food source. Polysaccharide is the main active ingredient in *G. elata*, which has pharmacological activities such as immune regulation, anti-oxidation, anti-cancer, anti-aging, neuroprotection and antibacterial activity and so on. The biological activities of *G. elata* polysaccharide (GPs) is closely related to its chemical structures. However, no a review has synthetically summarized the chemical structures and pharmacological activities of GPs. This study delves into the chemical structures, pharmacological action of GPs, offering insights for the future development an application of these compounds.

## 1 Introduction

Gastrodiae Rhizoma (known as *Tianma* in China) is the dry tubers of *G. elata* Blume (*G. elata*), which was first mentioned in the *Shen Nong’s Herbal Classic* and was widely distributed in in Sichuan, Guangdong, Yunnan and Guizhou provinces (Wang et al., 2022). According to the theory of Traditional Chinese Medicine (TCM), *G. elata* nature is naturally warm and tastes sweet, returns to the liver meridian, which has the function of calming wind and stopping convulsive seizures, suppressing liver yang, expelling wind and clearing collateral. In clinical practice, *G. elata* is widely used in the prevention and treatment of childhood convulsions, memory loss, sciatic neuropathy, epilepsy and other diseases, and is also widely used in health products and food fields (Zhang et al., 2007). Modern pharmacology recognizes that *G. elata* and its extracts have anti-tumor, anti-oxidation and anti-aging effects, regulating immunity, sedation, hypoglycemia, hypolipidemia, anti-depression, anti-viral, and anti-convulsant effects (Liu and Huang, 2017).

Studies have shown that 134 bioactive compounds originate from *G*. *elata*, including phenolic compounds, polysaccharides, organic acids and sterols (Feng et al., 1979; Yang et al., 2007; Duan et al., 2013; Zhu et al., 2019). Some of these molecules showed activity against migraines, hypertension, and other neurological diseases (Hayashi et al., 2002; Zhu et al., 2019). It has been suggested that *G. elata* polysaccharides (GPs) are active compounds with a wide range of pharmacological effects, such as anti-oxidant, anti-cancer, anti-virus, anti-osteoporosis, immunomodulatory, and neuroprotective effects and so on (Qiu et al., 2007; Chen et al., 2015; Liu and Mori, 1992; Liu et al., 2015; Bao et al., 2017). Due to its great medical and health value, more and more researchers are paying attention to the pharmacological activities of GPs. Furthermore, many studies have attested that the biological activities of GPs are closely related to their chemical structures. However, no previous articles have synthetically summarized the chemical structures and pharmacological activities of GPs. In this article, we review the structural characteristics, biological activities and structure-activity relationships of GPs, to aid in providing a theoretical basis and data for the research, development and utilization of GPs.

## 2 The structural features of GPs

**FIGURE 1:**
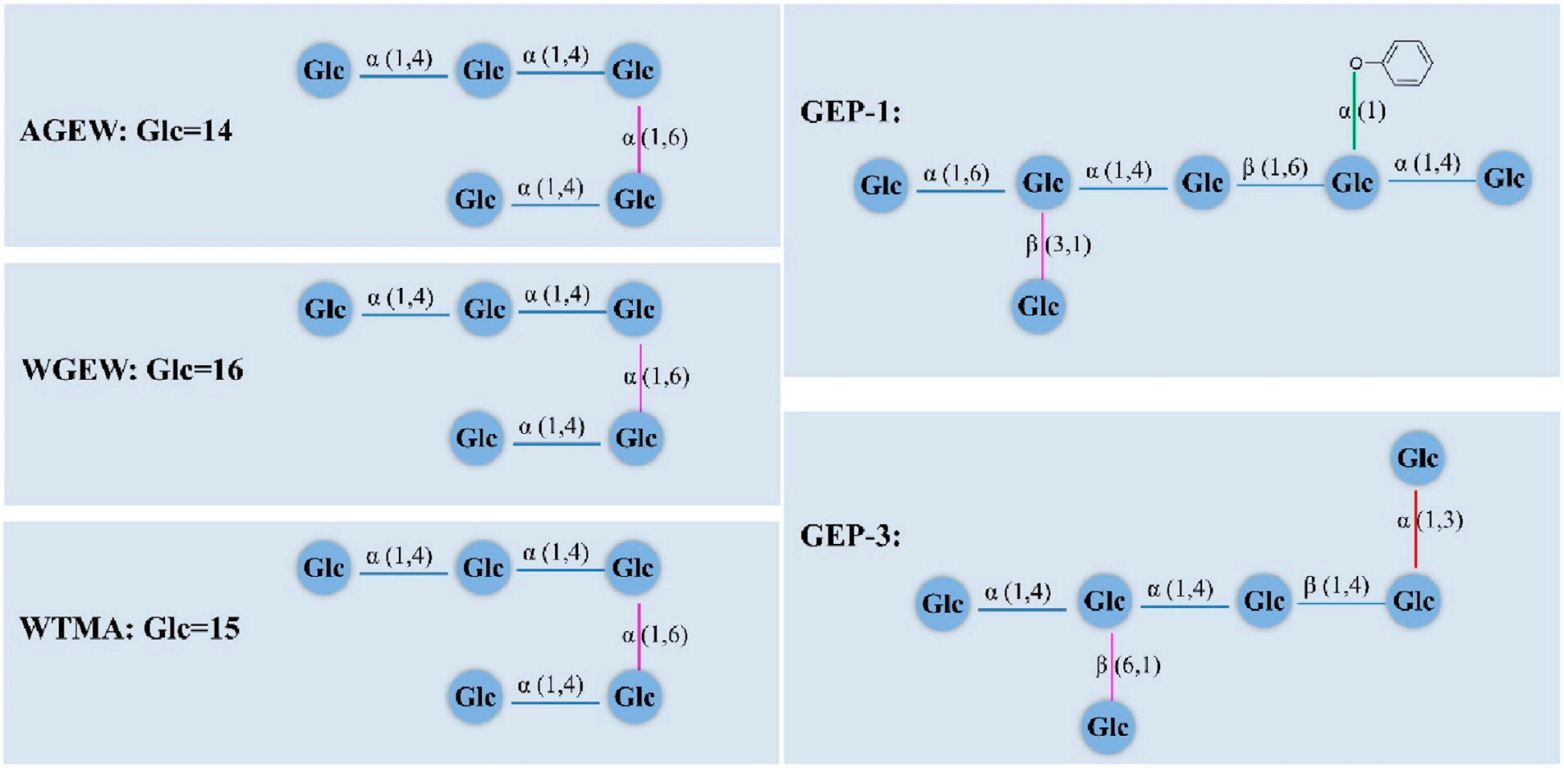
Structures of GPs compounds in *G. elata.*

The structures of polysaccharides can be divided into primary structure and high-level structure. The primary structure includes molecular weight, monosaccharide composition, glycosidic bond configuration, repeating structural units and branching degree. The high-level structure (secondary, tertiary and quaternary structures) is mainly the spatial conformation of polysaccharides (Zhang et al., 2018). To date, more than 20 GPs with known structures have been extracted and separated. The primary structural characteristics of the GPs, including molecular weight, monosaccharide composition, molar ratio, and backbone, are summarized in Table 1. The structures of the some GPs are shown in Figure 1.

**TABLE 1:** The chemical structures of *Gastrodia elata* Blume polysaccharides.

Compound name	Molecular weight (Da)	Monosaccharide composition and molar ratio	Backbone	Ref.
**WGEW**	1.00 × 10^5^	Glc	α-1,4-Glcp α-1,4,6-Glcp	Qiu et al. (2007)
**AGEW**	2.80 × 10^5^	Glc	α-1,4-Glcp α-1,4,6-Glcp	Qiu et al. (2007)
**GPs**	2.71 × 10^5^	Glc	α-1,4-Glcp	Bao et al. (2017)
**GPSa**	4.97 × 10^5^	Rha: Man: Glc: 1: 1.07: 67.24	α-1,4-Glcp	Zhu et al. (2010)
**WTMA**	7.00 × 10^5^	Glc	α-1,4-Glcp α-1,4,6-Glcp	Chen et al. (2011)
**PGEB-3H**	2.88 × 10^4^	Glc	α-1,4-Glcp α-1,4,6-Glcp	Ming et al. (2012)
**Acidic polysaccharides**	–	Xyl: Glc: GlcA: GlaA	–	Lee et al. (2012)
**RGP-1a**	1.93 × 10^4^	Glc: Fru: 10.68: 1	–	Chen et al. (2016)
**RGP-1b**	3.92 × 10^3^	Glc	–	
**PGE**	1.54 × 10^6^	Glc	α-1,4-Glcp α-1,4,6-Glcp α-1,3-Glcp	Zhu et al. (2018)
**GEP**	8.75 × 10^6^	Glc	–	Chen et al. (2018a)
**GEP-3**	2.52 × 10^4^	Glc	α-1,4-Glcp β-1,4-Glcp β-1,6-Glcp α-1,3,4-Glcp	Huo et al. (2021)
**GEP-1**	2.01 × 10^5^	Glc	α-1,4-Glcp α-1,4,6-Glcp β-1,6-Glcp β-1,3-Glcp p-hydroxybenzyl alcoho	Huo et al. (2021)
**GEP-1**	7.64 × 10^4^	Ara: Gal: Glc: Man: 2.189: 4.791: 92.035: 0.342	α-1,4-Glcp	Guan et al. (2022)
**GEPs**	2.90 × 10^5^	Glc: Gal: GlcA: 88.21: 4.48: 4.40	α-1,4-Glcp	Li N. et al. (2023)
**GaE-B**	2.15 × 10^5^	Man: Rha: Glc: Gal: Xyl: 5.36: 2.64: 77.35: 5.33: 9.34	–	Ji et al. (2022)
**GaE-R**	1.49 × 10^5^	Man: Rha: Glc: Gal: Xyl: 5.07: 3.18: 71.01: 6.41: 14.32	–	Ji et al. (2022)
**GaE-Hyb**	1.95 × 10^5^	Man: Rha: Glc: Gal: Xyl: 4.83: 3.02: 77.58: 4.76: 9.81	–	Ji et al. (2022)
**GaE-G**	2.51 × 10^5^	Man: Rha: Glc: Gal: Xyl: 3.64: 2.96: 81.88: 3.11: 8.40	–	Ji et al. (2022)
**GEP2-6**	2.71 × 10^6^	Glc	α-1,4-Glcp α-1,6-Glcp	Chen et al. (2024)

Notes:–Indicates that the item is not detected; Glc: glucose, Man: mannose, Rha: rhamnose, Gal: galactose, Xyl: xylose, Fru: fructose, GlcA: glucuronic acid, GlaA: galacturonic acid.

Qiu et al. (2007) obtained two glucans (WGEW and AGEW) from *G. elata* Blume., with molecular weight of AGEW and WGEW was 2.80 × 10^5^ Da and 1.00 ×·10^5^ Da, respectively. Their structures have an α-(1→4)-linked glucosyl backbone. Methylation analysis showed that two polysaccharides have terminal Glc, 1,4- and 1,4,6-linked Glc, the ratio of Glc:1,4-:1,4,6-linked Glc in WGEW was 1:16:1, and the ratio of it in AGEW was 1: 14: 1. Zhu et al. (2010) obtained *G.elata* polysaccharide (GPSa), with a molecular weight of 4.97 × 10^5^ Da. Structural analysis revealed that GPSa was composed mainly of glucose, but also contained small amounts of rhamnose and mannose. The molar ratio of GPSa is rhamnose: mannose: glucose: 1: 1.07: 67.24. IR and NMR analysis indicated GPSa chain was α-(1→4) glucan with α-(1→4) glucosyl branches. Chen et al. (2011) also obtained water-soluble glucan (WTMA) from the rhizome of Gastrodia elata Bl. The mean molecular weight of WTMA was 7.0 × 10^5^ Da, with the results showed that WTMA was an α-(1→4) glucan with α-(1→4) glucosyl branches attached to O-6 of the branch points. Ming et al. (2012) purified *G. elata* polysaccharide (PGEB-3H), was found to be a glucan with a molecular weight of 2.88 × 10^4^ Da. Structural analysis showed that PGEB-3H was consisted of 1,4-linked glucose and 1,4,6-linked glucose with an approximate molar ratio of 20: 1. FT-IR analysis indicated a pyranose form of the glucosyl residue, absorption at 1027.0 cm^−1^, 1079.6 cm^−1^, and 1153.2 cm^−1^. Lee et al. (2012) obtained an acidic polysaccharide. It was purified from the crude polysaccharides by DEAE-Sepharose CL-6B. The analysis was shown that the fraction of acidic polysaccharide included xylose, glucose, galacturonic acid, and glucuronic acid (Table 1). Chen et al. (2016) separated two homogeneous polysaccharides (RGP-1a and RGP-1b) from the residue of Rhizoma gastrodiae. The results showed that RGP-1a was composed of fructose and glucose in a molar ratio of 1:10.68, and RGP-1b was mainly consisted of glucose. Bao et al. (2017) obtained a homogeneous polysaccharide (GPs), with a molecular weight of 2.71 × 10^5^ Da. Analysis of the monosaccharide composition of GPs showed that GPs was composed of glucose. Zhu et al. (2018) yielded a polysaccharide (PGE) with hot water and purified it with Sephadex G-200 followed by ultra-filtration. This study indicated that PGE had a molecular weight of 1.54 × 10^6^ Da, the backbone of PGE composed of (1→4)-linked-D-Glcp and the branches are (1→3)-linked-D-Glcp, (1→4,6)-linked-d-Glcp and (1→)-linked-glucose terminal. Further detailed data are shown in Table 1. Chen et al. (2018a) isolated a *G. elata* Blume polysaccharide (GEP), with a molecular weight of 8.75 × 10^6^ Da. IR and NMR showed that GEP was consists of glucose. Huo et al. (2018) obtained a homogeneous polysaccharide which was named GEP-1. It was isolated and purified from *G. elata* by hot-water extraction, ethanol precipitation, and membrane separator. The structural analysis showed that the backbone of GEP-1 consisted of 1,3,6-linked-α-Glcp, 1,4-linked-α-Glcp, 1,4-linked-α-Glcp and 1,4,6-linked-α-Glcp, with a molecular weight of 2.01 × 10^5^ Da, and contained a citric acid and repeating the p-hydroxybenzyl alcohol as a branch. Guan et al. (2022) isolated a polysaccharide from *G. elata* (named GEP-1), with a molecular weight of 7.64 × 10^5^ Da. NMR and methylation analyses revealed that the main chain structure of GEP-1 was α-(1→4)-glucans. Li F. et al. (2013) obtained a polysaccharide named GEPs, with a molecular weight of 2.92 × 10^5^ Da, which consists of glucose, galactose and galacturonic acid was in the ratio of 88.21: 4.48: 4.40. Ji et al. (2022) obtained four components of GaE-B (*G. elata Bl. f. glauca S. chow* polysaccharides), GaE-R (*G. elata Bl. f. elata* polysaccharides), GaE-Hyb (*hybridization of G. elata Bl. f. glauca S. chow and G. elata Bl. f. elata* polysaccharides), and GaE-G (*G. elata Bl. f. viridis Makino* polysaccharides). Based on HPGPC analysis, their average molecular weight are 2.15 × 10^5^ Da, 1.49 × 10^5^ Da, 1.95 × 10^5^ Da, 2.51 × 10^5^ Da, respectively. GC analysis showed that these GaE polysaccharides were heteropolysaccharides, and the polysaccharides comprised Man, Rha, Glc, Gal, and Xyl. The detail more ratio shown in Table 1. Chen et al. (2024) obtained a water-soluble polysaccharide (GEP2-6), with a molecular weight of 2.71 × 10^6^ Da, which consists of only glucose. NMR and methylation analyses revealed that the main chain structure of GEP2-6 was consists of α-(1→4) and α-(1→6) glycosidic bonds.

## 3 Biological activities

**FIGURE 2:**
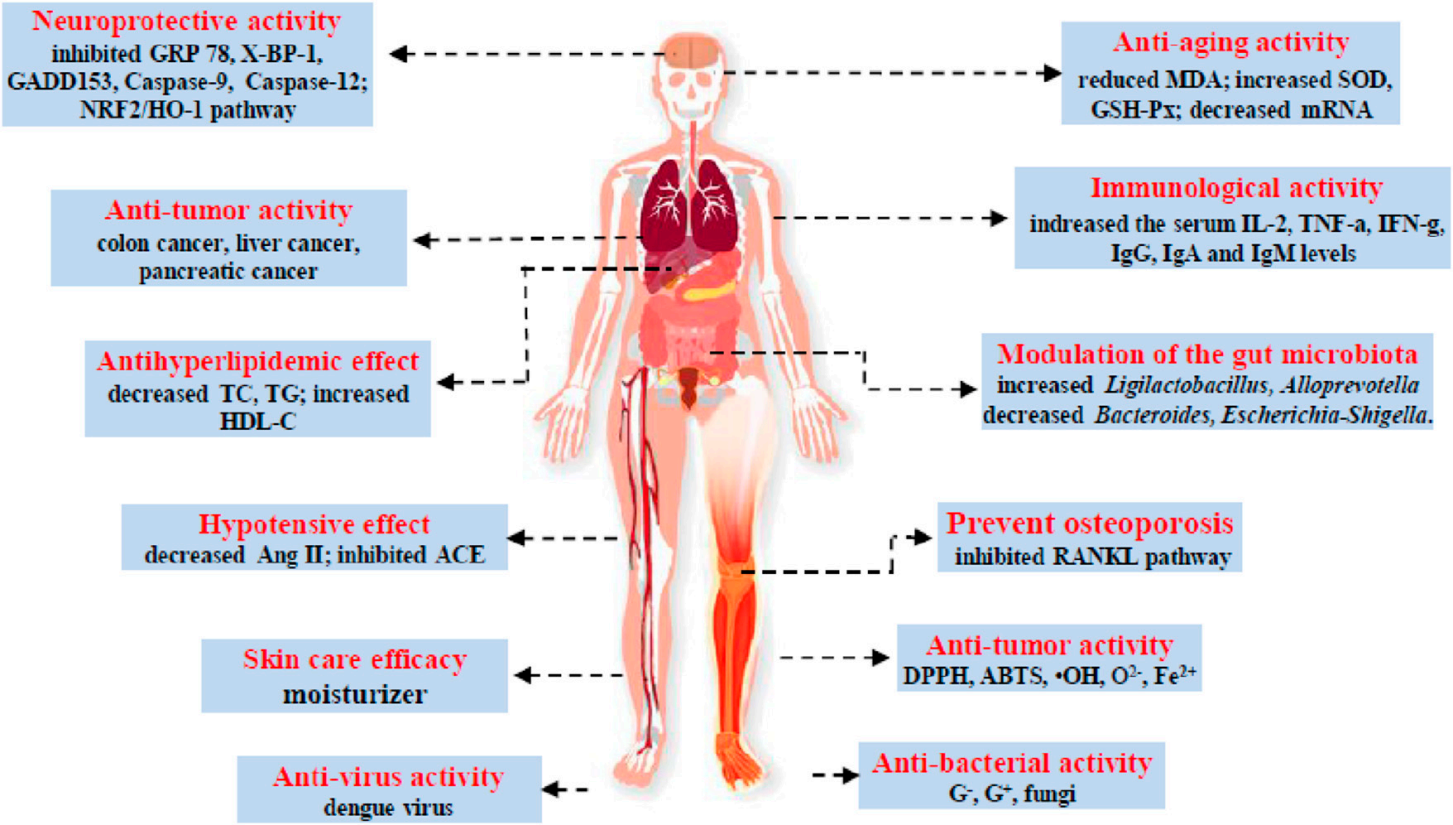
The health functions of GPs.

In recent years, research has focused on the pharmacodynamics of GPs. Many references point out that GPs showed that significant pharmacological activies, sush as anti-oxidation, anti-tumor, immune regulation, anti-aging, improve memory, improve cerebral ischemia, reduce blood pressure, anti-bacterial effect and reduce blood lipid (Figure 2) (Zhu et al., 2019; Wang et al., 2022). The biological activities of GPs are summarized in Table 2.

**TABLE 2:** Biological activities of GPs isolated from the *Gastrodia elata*.

Biological activies	Name	Description	*In vivo*/*In vitro*	Ref.
Anti-oxidative activity	GP	evaluated the scavenging activity of DPPH and ABTS.	*In vitro*	Hou and Hou (2018)
	heteropolysaccharides	tested the activites of DPPH radicals, ABTS radicals, superoxide radicals, hydroxyl radicals, ferrous ion chelating capacity, and reducing power	*In vitro*	Ji et al. (2022)
	GPs	The scavenging rate of DPPH and ABTS was higher, and the antioxidant capacity was lower than that of Vc	*In vitro*	Wang et al. (2022)
	GEP1-G GEP2-G	The clearance rates of DPPH were 44.5% and 25.6%, the clearance rates of O^2-^· were 33.32% and 21.55%, the clearance rates of ·OH were 39.5% and 22.8%	*In vitro*	Chen et al. (2018b)
	GPs	the clearance rate of DPPH and ·OH was 40.52% and 36.52%	*In vitro*	Zhang et al. (2021)
	GPs	has the best removal effect on hydrogen peroxide (H_2_O_2_), the clearance rates was 25.80%	*In vitro*	Xu et al. (2015)
	GPs	the concentration IC_50_ were 1.18 mg/mL (·OH), 1.62 mg/mL (O^2-^·)	*In vitro*	Liu et al. (2009)
	GPs	has a certain scavenging effect on ferrous ions, ABTS free radicals, hydroxyl free radicals and DPPH free radicals	*In vitro*	Zhang et al. (2019)
	GEP2-6	scavenged DPPH and hydroxyl radicals	*In vitro*	Chen et al. (2024)
Anti-aging activity	GEP	reduced the MDA level, increased the SOD and GSH-Px activities	*In vivo*	Chen et al. (2018c)
	GPs	increased SOD and GSH-Px activity and decreased MDA and NO content	*In vivo*	Li F. et al. (2013)
	GPs	related to oxidative metabolism in the body	*In vivo*	Xie et al. (2010)
	GPs	increased the activities of SOD and CAT in serum, liver, brain and heart	*In vivo*	Kong et al. (2005)
	GPs	decreased the mRNA expression and protein level of caspase-3, MURF-1 and MAFbX	*In vivo*	Wang et al. (2019)
Anti-tumor activity	WTMA	inhibited PANC-1 cell growth, showed no effect on PANC-1 cells growth	*In vitro*	Chen et al. (2011)
	GPs	inhibited at 90 mg/kg, and the inhibition rate was 27.6%	*In vitro*	Wang et al. (2014)
	GPs	increased G0/G1 phase and decrease G2/M phase	*In vitro*	Liu et al. (2015)
	WSS25	blocked of BMP/Smad signaling pathway	*In vitro*	Qiu et al. (2010)
	PGEs	promoted late apoptosis and arrested at G2/M phase	*In vitro*	Dai et al. (2021)
Immunological activity	RGP-1a RGP-1b	effected the NO production and phagocytic activity	*In vitro*	Chen et al. (2016)
	GPs	indreased the serum IL-2, TNF-a, IFN-g, IgG, IgA, IgM levels, and the spleen and thymus indexes	*In vivo*	Bao et al. (2017)
	GEP-1	induced TNF-α, IL1-β and NO release	*In vitro*	Guan et al. (2022)
	GEPs	increased content of SCFAs	*In vitro*	Li N. et al. (2023)
	GPs	regulated the levels of IgA, IgG, IgM and hemolysin in mice, increased the index of thymus and spleen	*In vitro* *In vivo*	Dai et al. (2021)
	GPs	reduced the activity of ALT, AST, NO and the contents of TNF-α and IL-1 in serum of mice, inhibited MAD, increased SOD.	*In vitro* *In vivo*	Li et al. (2015)
	GPs	stimulated IL-2, TNF-α, IFN-γ, IgG, IgA and IgM	*In vivo*	Li et al. (2016)
Neuroprotective activity	GPs	decreased BCL-12 and BAX protein, inhibited the expression of caspase-3 protein	*In vitro*	Zhou et al. (2013)
	GPs	reduced the level of intracellular toxic reactive oxygen species, reduced the release of LDH, inhibited the expression of GRP 78, X-BP-1, GADD153, caspase-9 and caspase-12	*In vitro*	Zhou et al. (2017)
	NPGE	attenuated ferroptosis-mediated neuroinflammation via the NRF2/HO-1 signaling pathway	*In vitro*	Zhang et al. (2023)
	GPs	increased Bcl-2 expression in brain tissue, reduced the expression of Bax	*In vitro*	Wang et al. (2019)
Hypotensive effects	GPs	reduced systolic blood pressure in SHR fed a high-fat diet	*In vitro*	Lee et al. (2012)
	PGE	exhibited ACE-inhibitory activity	*In vitro*	Zhu et al. (2018)
	GPs	decreased the levels of Ang II, and increased the levels of NO were increased	*In vitro*	Wang et al. (2019)
Antihyperlipidemic effects	PGEB-3H	caused 29% increase in HDL-C	*In vitro*	Ming et al. (2012)
	GPs	decreased hypolipidemic indexes (total cholesterol, triglyceride and low-density lipoprotein cholesterol levels)	*In vivo*	Lee et al. (2012)
	PGEB-3-H	decreased the content of TC and TG and increased HDL-C, had no significant effect on the content of LDL-C	*In vitro*	Miao and Shen (2006)

### 3.1 Anti-oxidation activities

Free radicals can accelerate the oxidation process *in vivo* and lead to cell aging. Previous studies have shown that GPs can effectively remove free radicals including 1,1-diphenyl-2-picrylhydrazyl (DPPH), oxygen radicals (O^2-^·), and hydroxyl radicals (·OH). GPs has good antioxidant activity, as evaluated by DPPH, O^2-^·and·OH assays. The clearance rate of DPPH, O^2-^·and OH was around 50%, when the concentration of GPs was 1–3.5 mg/mL (Hou and Hou, 2018; Chen et al., 2018b; Zhang et al., 2021; Chen et al., 2024; Liu et al., 2009; Wang, et al., 2022). Xu et al. (2015) reported that GPs had the best removal effect on hydrogen peroxide (H_2_O_2_), the clearance rates was 25.80%, and the scavenging power of other free radicals as following DPPH (22.37%) > ONOO^−^ (20.52%) > O^2-^ (12.23%) > -OH (4.85%). Chen et al. (2018a) found GEP had high radical-scavenging activities. At concentration of 200 mg/mL, the HRSA and DRSA of the GEP were 94.56% and 84.21%, respectively. In addition, GPs have a strong scavenging effects on ABTS radicals, superoxide radicals, ferrous ion chelating capacity, and reducing power (Hou and Hou, 2018; Zhang et al., 2019; Ji et al., 2022; Wang, et al., 2022). The above studies showed that GPs had a strong antioxidant effect. The antioxidant range of heteropolysaccharides is wider than that of glucan from *G. elata*.

### 3.2 Anti-aging activities

Many studies have shown that GPs can improve the expression of peroxidase and slow down the aging of organs and tissue. Li N. et al. (2023) reported that GPs had anti-aging effects in D-galactose-induced senescence mice. GPs significantly increased SOD and GSH-Px activity and decreased MDA and NO contents in aging mice, and showed a good dose-dependent relationship. Xie et al. (2010) found that GPs can improve the learning and memory ability of D-galactose-induced aging mice, its mechanism is mainly related to oxidative metabolism in the body. The finding of Kong et al. (2005) displayed that GPs significantly increased the activities of SOD and CAT in the serum, liver, brain and heart tissue of aging mice, significantly inhibited the formation of MDA in the serum, liver, brain and heart tissue of aging mice, and significantly increased the activity of GSH-Px in the serum of aging mice. The results indicated that GPs had better scavenging free radicals, decreasing MDA content and delaying cell aging. Chen et al. (2018b) found that intragastric administration of GEP significantly decreased the MDA levels but significantly increased SOD and GSH-Px activities in the sera and brains of D-galactose-induced aging mice as compared with those of the model group, indicated that GEP can effectively suppress oxidation-induced damage to the sera and brain tissues of D-galactose-induced aging mice. Wang and Liu (2019) found that GPs could delay skeletal muscle aging in mice by reducing the mRNA expression and protein levels of caspase-3, MURF-1 and MAFbX in muscle tissue. However, the molecular mechanism of anti-aging is not been clarified.

### 3.3 Anti-tumor activities

**FIGURE 3:**
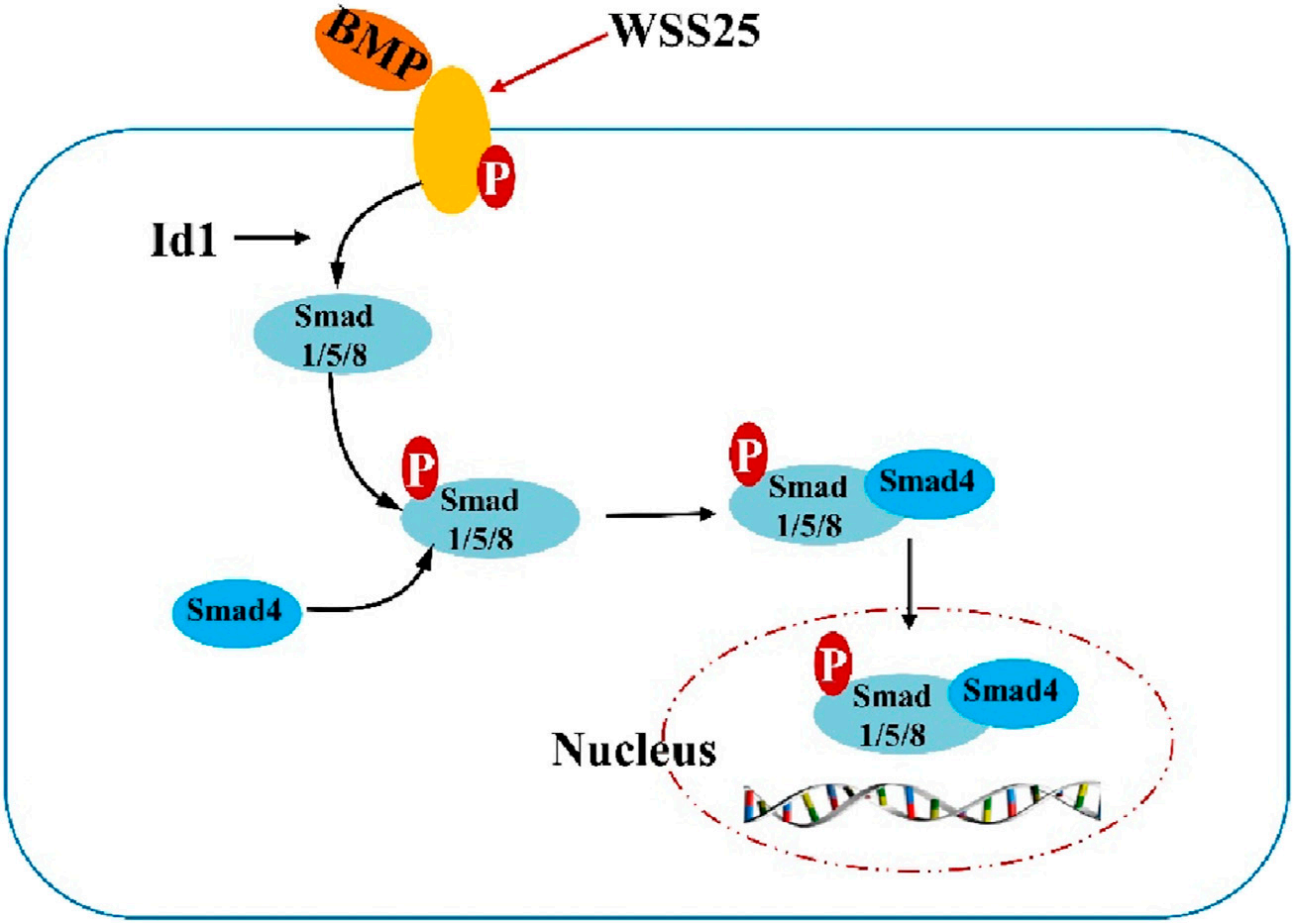
The mechanism of WSS25 in hepatocellular cancer cell lines.

Numerous cell and animal model studies have shown that GPs can significantly inhibit the development of various types of cancer, such as colon cancer, liver cancer, pancreatic cancer, etc. Wang et al. (2014) found that the tumor growth of GPs was significantly inhibited at 90 mg/kg, and the inhibition rate was 27.6%. Liu et al. (2015) reported that GPs have a significant anti-cancer effect on H22 tumor-bearing mice, the results showed that the GPs inhibition rate on H22 cells was 44.7%. The mechanism is mainly related to GPs could increase the cell percentage in the G0/G1 phase and decrease cell percentage in the G2/M phase. Qiu et al. (2010) reported that WSS25 could inhibit the growth of xenografted hepatocellular cancer cells in nude mice, its mechanism is related to the blocking of BMP/Smad signaling by WSS25, as shown in Figure 3. Dai et al. (2021) investigated the anti-tumor activities of *G*. *elata* polysaccharides (PGEs) against MCF-7 cells *in vitro*. The results showed that the PGEs could inhibit the growth of MCF-7 cells by promoting late apoptosis and arresting at G2/M phase. Chen et al. (2011) investigated the anti-pancreatic cancer activities of WTMA against PANC-1 cell lines and showed no effect on the growth of PANC-1 cells.

### 3.4 Immunological activities

Numerous *in vitro* and *in vivo* studies have demonstrated the immunological activities of GPs. Li et al. (2016) found that GPs can regulate the levels of immunoglobulin (IgA, IgG, IgM) and hemolysin in mice, and increase the index of thymus and spleen. Li et al. (2015) reported that GPs significantly reduced the activity of ALT, AST, NO and the content of TNF-α and IL-1 in the serum of mice, inhibited the level of MAD in the liver, increased the activity of SOD and the concentration could significantly increase the proliferation ability of T and B lymphocytes in the spleen. The results indicated that GPs had a good protective effect against immunological liver injury in mice. Li F. et al. (2013) found that GEPs can effectively alleviate immunosuppression, the potential mechanism was related to the modulation of gut microbiota composition by GEPs and the resulting increased content of SCFAs. Chen et al. (2016) found that the two polysaccharides (RGP-1a and RGP-1b) have a significant impact on NO production and phagocytic activity of RAW264.7 macrophages. Compared to RGP-1a, RGP-1b, which has a smaller molecular weight and a uniform monosaccharide composition, exhibits superior immunological activities in RAW264.7 macrophages. Molecular weight and homogeneous composition may be key factors affecting the immunological activity of GPs. Bao et al. (2017) found that GPs can increase serum IL-2, TNF-α, IFN-g, IgG, IgA and IgM levels, as well as spleen and thymus indices of Kunming mice, showing that GPs could improve the immune function of immunosuppression model mice. Guan et al. (2022) observed the effect of GEP-1 on immune function by increasing phagocytic activities and induced release of cytokines (TNF-α, IL1-β) and nitric oxide (NO) in macrophages.

### 3.5 Neuroprotective activities

**FIGURE 4:**
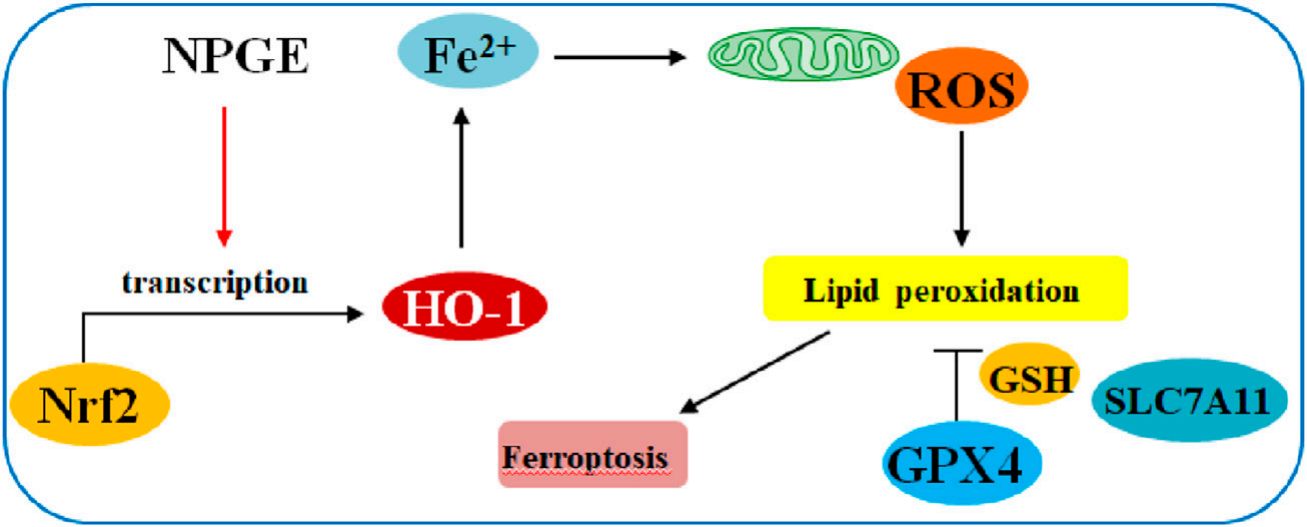
Schematic illustration of NPGE in BC cells through of the NRF2/HO-1 pathway.

The neuroprotective effect of GPs on rat pheochromocytoma nerve cells (PC12) has recently attracted great attention. Zhou et al. (2013) found that GPs significantly could improve corticosterone (CORT)-induced injury and cell morphology of PC12 cells, reduce the expression of BCL-12 and BAX protein, and inhibit the expression of caspase-3 protein. Zhou et al. (2017) reported that GPs play a protective role in nerve cells by reducing the level of intracellular toxic reactive oxygen species, reducing the release of LDH, and inhibiting the expression of GRP 78, X-BP-1, GADD153, caspase-9 and caspase-12. Zhang et al. (2023) reported that neutral polysaccharide from *G. elata* (NPGE) had potential effects on the neuropathology of cerebral ischemia-reperfusion injury (CIRI). Its mechanism is related to that NPGE alleviates CIRI by attenuating ferroptosis-mediated neuroinflammation via the NRF2/HO-1 signaling pathway, the relevant mechanism is shown in Figure 4. In addition, GPs could increase the expression of anti-apoptotic gene Bcl-2 in brain tissue reduce expression of apoptosis gene Bax, alleviating cerebral palsy, apoptosis of brain tissue, exerting neuroprotective activity (Wang et al., 2019).

### 3.6 Hypotensive effects

Numerous studies have demonstrated the blood pressure lowering effect of GPs. Angiotensin-converting enzyme (ACE) plays a significant role in the development of hypertension in the body. Miao and Shen (2006) observed the effect of GPs on angiotensin Ⅱ (Ang Ⅱ) level, the results showed that Ang II levels were decreased and the NO levels were increased. Zhu et al. (2018) found that PGE had ACE inhibitory activity, the inhibition rate of PGE on ACE was calculated to be 74.40% and the IC_50_ value was 0.66 mg/mL. Lee et al. (2012) reported that the acidic polysaccharide fraction from Gastrodia rhizome significantly reduced blood pressure in SHR fed a high-fat diet.

### 3.7 Antihyperlipidemic effect

Ming et al. (2012) reported effects of PGEB-3-H on total cholesterol (TC), triglycerides (TG), low-density lipoprotein cholesterol (LDL-C) and high-density lipoprotein cholesterol (HDL-C). The results showed that PGEB-3-H could reduce the content of TC and TG and increase the level of HDL-C, but had no significant effect on the LDL-C content. It can be seen that PGEB-3-H has a potential effect on lowering blood lipids and is related to the regulation of cholesterol content. Lee et al. (2012) studies showed that the hypolipidemic indexes (total cholesterol, triglyceride and low-density lipoprotein cholesterol levels) of the acidic polysaccharide groups were lower than those in the control group. These results indicated that acidic polysaccharide improve serum lipid levels.

### 3.8 Other activities

GPs has various structures and diverse pharmacological effects. A large number of studies have shown that GPs play an effective role in anti-bacterial activity, osteoporosis prevention, liver protective effects, memory improvement and skin care effectiveness. Chen et al. (2018c) found that GPs had an inhibitory effect on G^−^, G^+^ and fungi. Chen et al. (2015) investigated that a sulfated polysaccharide (WSS25) extracted from the rhizome of *G. elata* inhibited RANKL-induced osteoclast formation in RAW264.7 cells and BMMs by blocking the BMP-2/Smad/Id1 signaling pathway. Shi et al. (2017) reported that GPs could improve the memory of rats with cerebral palsy by regulating neurotransmitter in the brain. A number of studies have applied GPs to develop a skin care product (Wang et al., 2016; Du and Chen, 2018; Zheng et al., 2018). Qiu et al. (2007) reported that WGEW and AGEW showed strong anti-dengue virus bioactivity. Chen et al. (2024) found that four heteropolysaccharides had an inhibitory effect on the anti-hyperglycaemic activity of α-amylase and α-glucosidase. Xu et al. (2023) reported that GPs had modulation of gut microbiota and improvement in metabolic disorders.

## 4 Conclusion

In conclusion, as a traditional Chinese medicine, *G. elata* is widely used in medicine, food and health products. *G. elata* polysaccharides are one of the main components of *G. elata*. Due to its pharmacological effects such as anti-oxidation, anti-tumor, immune regulation and memory improvement, it has attracted great attention from scientists in medicine and healthcare fields. In this paper, structural analysis and pharmacological activities of related research, further study of *G. elata* polysaccharides and rational application for reference.
